# Mesenchymal stem cell therapy ameliorates diabetic nephropathy via the paracrine effect of renal trophic factors including exosomes

**DOI:** 10.1038/srep34842

**Published:** 2016-10-10

**Authors:** Kanna Nagaishi, Yuka Mizue, Takako Chikenji, Miho Otani, Masako Nakano, Naoto Konari, Mineko Fujimiya

**Affiliations:** 1 Second Department of Anatomy, Sapporo Medical University, Japan; 2 Department of Diabetic Cellular Therapeutics, Sapporo Medical University, Japan

## Abstract

Bone marrow-derived mesenchymal stem cells (MSCs) have contributed to the improvement of diabetic nephropathy (DN); however, the actual mediator of this effect and its role has not been characterized thoroughly. We investigated the effects of MSC therapy on DN, focusing on the paracrine effect of renal trophic factors, including exosomes secreted by MSCs. MSCs and MSC-conditioned medium (MSC-CM) as renal trophic factors were administered in parallel to high-fat diet (HFD)-induced type 2 diabetic mice and streptozotocin (STZ)-induced insulin-deficient diabetic mice. Both therapies showed approximately equivalent curative effects, as each inhibited the exacerbation of albuminuria. They also suppressed the excessive infiltration of BMDCs into the kidney by regulating the expression of the adhesion molecule ICAM-1. Proinflammatory cytokine expression (e.g., TNF-α) and fibrosis in tubular interstitium were inhibited. TGF-β1 expression was down-regulated and tight junction protein expression (e.g., ZO-1) was maintained, which sequentially suppressed the epithelial-to-mesenchymal transition of tubular epithelial cells (TECs). Exosomes purified from MSC-CM exerted an anti-apoptotic effect and protected tight junction structure in TECs. The increase of glomerular mesangium substrate was inhibited in HFD-diabetic mice. MSC therapy is a promising tool to prevent DN via the paracrine effect of renal trophic factors including exosomes due to its multifactorial action.

Diabetic nephropathy (DN) is a microvascular complication of diabetes and the most common cause of end-stage chronic kidney disease, accounting for the cardiovascular complications and high mortality rate of patients with diabetes^1^,^2^,^3^. The development of DN is stimulated by sustained hyperglycemia^4^. High levels of blood glucose induce oxidative stress and inflammation, which are closely linked with the development of DN^5^,^6^. Higher levels of reactive oxygen species can induce the production of inflammatory cytokines in the kidney, which enhances DN progression^7^. One of the sources of inflammatory cytokines is bone marrow-derived cells (BMDCs). Previously, we demonstrated that BMDCs excessively infiltrate a kidney fused with tubular epithelial cells (TECs), causing parenchymal cells to produce cytotoxic tumor necrosis factor alpha (TNF-α) and caspase-3, which leads to the degeneration and apoptosis of TECs in streptozotocin (STZ)-induced and high-fat diet (HFD)-induced diabetic mice^8^. On the other hand, Wise and Ricardo reported that cellular loss of renal tissue often leads to the infiltration of bone marrow-derived inflammatory cells; however, it is unclear whether this contributes to tissue destruction or repair of the extent of kidney injury^9^. Therefore, we examined the role of BMDC infiltration in the renal damage of diabetes by analyzing the changes due to therapeutic intervention.

Fibrosis is a major cause of the pathogenesis of DN. Myofibroblasts play a crucial role in the induction of fibrosis in the glomerulus and renal tubulointerstitium^10^. Epithelial-to-mesenchymal transition (EMT) is a major source of the myofibroblasts in proximal tubular cells and podocytes in DN^11^,^12^. Molecules associated with the transforming growth factor beta (TGF-β) superfamily are key regulators not only of EMT induction^13^,^14^ but also the synthesis of extracellular matrix molecules such as collagen type I, fibronectin, and laminin, leading to renal fibrosis^15^. Therefore, regulation of TGF-β is a potential therapeutic target for DN.

Therapies for DN have previously been limited to drugs for the improvement of blood pressure or blood glucose. Once a renal disorder progresses to an irreversible stage, hemodialysis is inevitable^16^. Thus, the development of novel therapeutic strategies is required urgently to preserve renal function in DN. Mesenchymal stem cells (MSCs) are present in the bone marrow and mesenchymal tissues, which have multipotent and self-renewing properties and can differentiate into cells of the mesodermal lineage^17^. The intravenous administration of bone marrow-derived MSCs is effective for DN^18^,^19^,^20^,^21^. MSCs can elicit the repair of damaged kidney by inhibiting the release of pro-inflammatory cytokines, suppressing inducible nitric oxide synthase, and promoting parenchymal cell proliferation^22^,^23^.

MSC therapy for DN is suggested to have a main mechanism of action via the various paracrine effects of trophic factors secreted by MSCs^24^. This hypothesis is reasonable since the number of MSCs in the kidney after systemic injection is very low in relation to therapeutic efficacy. In fact, systemic infusion of MSC-conditioned medium (MSC-CM) ameliorates podocyte apoptosis in the kidney of the STZ-induced type 1 diabetes (T1D) model^25^,^26^ and glomerular injury in a chronic kidney disease model^27^. Recently, microvesicles derived from MSCs, namely, exosomes, have been applied widely in experimental research and their contribution to the therapeutic effects of MSCs has been demonstrated^28^,^29^. Exosomes are known to be present in cell culture supernatants of MSCs. Exosomes contain proteins, mRNAs, DNA, and microRNAs (miRNAs)^30^, which contribute to the exchange of information between cells. Therefore, we focused on the role of exosomes as the key factor of the paracrine effects of MSCs in DN. Although the effect of exosomes on acute kidney disease has been reported^31^,^32^,^33^, the effectiveness of exosomes derived from bone marrow MSCs for DN, as a chronic renal disease, has not been clarified.

In this study, we investigated several effects of the intravenous administration of MSCs in the kidney of an HFD-induced type 2 diabetes (T2D) murine model and an STZ-injected insulin deficiency murine model as follows: (1) effect of MSC-CM containing exosomes in comparison to MSCs; (2) anti-inflammatory effect by inhibiting the hyperinfiltration of BMDCs; (3) anti-fibrotic action and protection of TECs from EMT; (4) anti-apoptotic effect and maintenance of epithelial barrier function in TECs; and (5) glomerular alterations in the HFD-induced T2D model. Our findings may open new prospects for the development of novel therapeutics for DN.

## Results

### MSC therapy prevents abnormal renal function induced by diabetes in HFD- and STZ-diabetic mice

**Figure 1:**
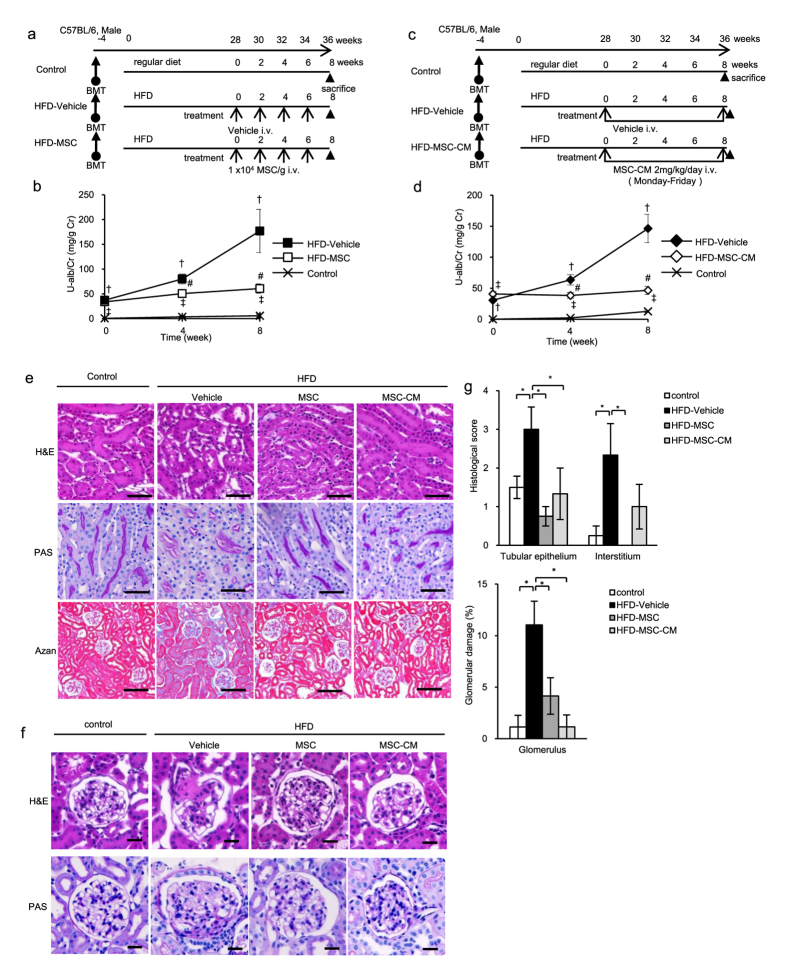
MSC and MSC-CM therapies for high-fat diet-induced diabetic mice. (**a**) Experimental protocol for MSC and (**c**) MSC-conditioned medium (MSC-CM) therapies in high-fat diet (HFD)-diabetic mice. (**b**) Changes in the albumin to creatinine ratio in urine (U-alb/Cr) after the initial administration of MSCs. (**d**) Changes in U-alb/Cr after the initial administration of MSC-CM. Data are expressed as mean ± SE values of 5–7 animals. ^†^*P* < 0.05 HFD-vehicle vs. control; ^‡^*P* < 0.05 HFD-MSC or HFD-MSC-CM vs. control; ^#^*P* < 0.05 HFD-MSC or HFD-MSC-CM vs. HFD-vehicle. (**e**) Histological findings of the renal cortex in H&E-, PAS-, and Azan-stained kidney sections at 8 weeks after initial MSC and MSC-CM therapies in HFD-diabetic mice. Bar: 50 μm in H&E and PAS staining; 100 μm in Azan staining. (**f**) Histological findings of glomeruli in H&E- and PAS-stained kidney sections at 8 weeks after initial MSC and MSC-CM therapies in HFD-diabetic mice. Bar: 20 μm in H&E and PAS staining. (**g**) Glomerular and tubular damage quantification in control, HFD-vehicle, HFD-MSC, and HFD-MSC-CM mice. Data shown are representative of 5 panels per animal at ×100 magnification. Data are expressed as mean ± SE of 3–5 animals.

**Figure 2:**
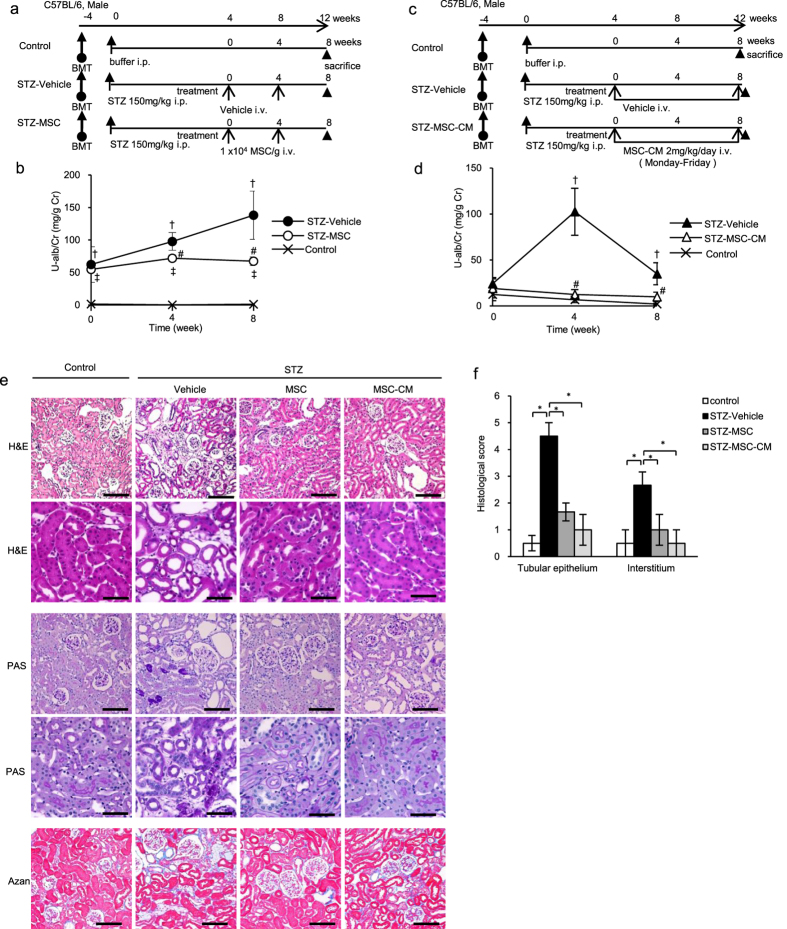
MSC and MSC-CM therapies for streptozotocin-induced diabetic mice. (**a**) Experimental protocol for MSC and (**c**) MSC-conditioned medium (MSC-CM) therapies in streptozotocin (STZ)-diabetic mice. (**b**) Changes in U-alb/Cr after the initial administration of MSCs. (**d**) Changes in U-alb/Cr after the initial administration of MSC-CM. Data are expressed as mean ± SE values of 5–7 animals. ^†^*P* < 0.05 STZ-vehicle vs. control; ^‡^*P* < 0.05 STZ-MSC vs. control; ^#^*P* < 0.05 STZ-MSC or STZ-MSC-CM vs. STZ-vehicle. (**e**) Histological findings of the renal cortex in H&E-, PAS-, and Azan-stained kidney sections at 8 weeks after initial MSC and MSC-CM therapies in STZ-diabetic mice. Bar: 100 μm in the upper panels of H&E, PAS, and Azan staining; 50 μm in the lower panels of H&E and PAS staining. (**f**) Glomerular and tubular damage quantification in control, STZ-vehicle, STZ-MSC, and STZ-MSC-CM mice. Data shown are representative of 5 panels per animal at ×100 magnification. Data are expressed as mean ± SE of 3–5 animals.

The experimental protocols for the MSC therapies in HFD- (Fig. 1a) and STZ-diabetic mice (Fig. 2a) are shown. Urine albumin excretion, which was estimated using the albumin to creatinine ratio (U-alb/Cr), was elevated significantly at 28 weeks after feeding an HFD to HFD-diabetic mice compared with age-matched control mice (Fig. 1b). U-alb/Cr was elevated significantly at 4 weeks after STZ administration to STZ-diabetic mice compared with control mice (Fig. 2b). MSC therapy inhibited the progression of diabetic renal failure, which was indicated by the inhibition of U-alb/Cr after 4 and 8 weeks of initial treatment in both HFD-MSC mice compared with HFD-vehicle mice (Fig. 1b) and STZ-MSC mice compared with STZ-vehicle mice (Fig. 2b). These effects were observed even though hyperglycemia was unchanged between STZ-vehicle and STZ-MSC mice or ameliorated slightly in HFD-MSC mice compared to HFD-vehicle mice at 8 weeks after the initial MSC treatment (Table 1).

### Small numbers of administered MSCs are detected in the kidney of HFD- and STZ-diabetic mice

The distribution of the administered MSCs was investigated in diabetic mice. Only a small number of PKH26-positive MSCs were detected in the tubular epithelial area of the kidney in HFD-MSC mice at 7 days after MSC administration (see Supplementary Fig. S2a). Green fluorescent protein (GFP)-positive MSCs were almost undetected in the kidney of STZ-MSC mice, while a small number of administered MSCs were detected in the spleen of STZ-MSC mice (see Supplementary Fig. S2b).

### MSC-CM therapy prevents abnormal renal function in HFD- and STZ-diabetic mice, similar to MSC therapy

The experimental protocol for MSC-CM therapies in HFD- (Fig. 1c) and STZ-diabetic mice (Fig. 2c) are shown. Similar to the effect of cell therapy, daily MSC-CM injections prevented the progression of renal failure, as indicated by the inhibition of U-alb/Cr after 4 and 8 weeks of treatment in both HFD-MSC-CM (Fig. 1d) and STZ-MSC-CM mice (Fig. 2d) compared with HFD-vehicle and STZ-vehicle mice, respectively. These effects were observed even though hyperglycemia was unchanged, similar to MSC therapy (Table 1).

### MSC and MSC-CM therapies prevent histopathological damage in the kidney of HFD- and STZ-diabetic mice

Morphological changes in the renal tubules of diabetic mice were observed. Vacuolation of the cytoplasm and disproportionation of nuclear polarity in TECs were detected in HFD-vehicle mice with hematoxylin and eosin (H&E) staining. The brush border was irregular and disrupted in the proximal tubules with periodic acid-Schiff (PAS) staining. Fibrotic changes were observed in the interstitial area of renal tubules and at the circumference of Bowman’s capsules with Azan staining (Fig. 1e). In addition, glomerular mesangial expansion was observed in HFD-vehicle mice with H&E and PAS staining (Fig. 1e,f).

Abnormal dilatation of the tubules, collective apoptosis in the tubular units, massive accumulation of inflammatory cells, and fibrotic changes in the interstitial area of the renal cortex were observed in STZ-vehicle mice with H&E, PAS, and Azan staining (Fig. 2e).

The quantitative value indicated that glomerulus damages were predominant in HFD mice, while degenerative changes in tubular epithelium and interstitial changes were characterized in STZ mice. MSC and MSC-CM therapies suppressed these pathological changes significantly in glomerulus, TECs and the interstitium, resulting in repair of the structure of renal tissue (Figs 1g and 2f).

### MSC and MSC-CM therapies suppress the excessive infiltration of BMDCs in the kidney of HFD- and STZ-diabetic mice

**Figure 3:**
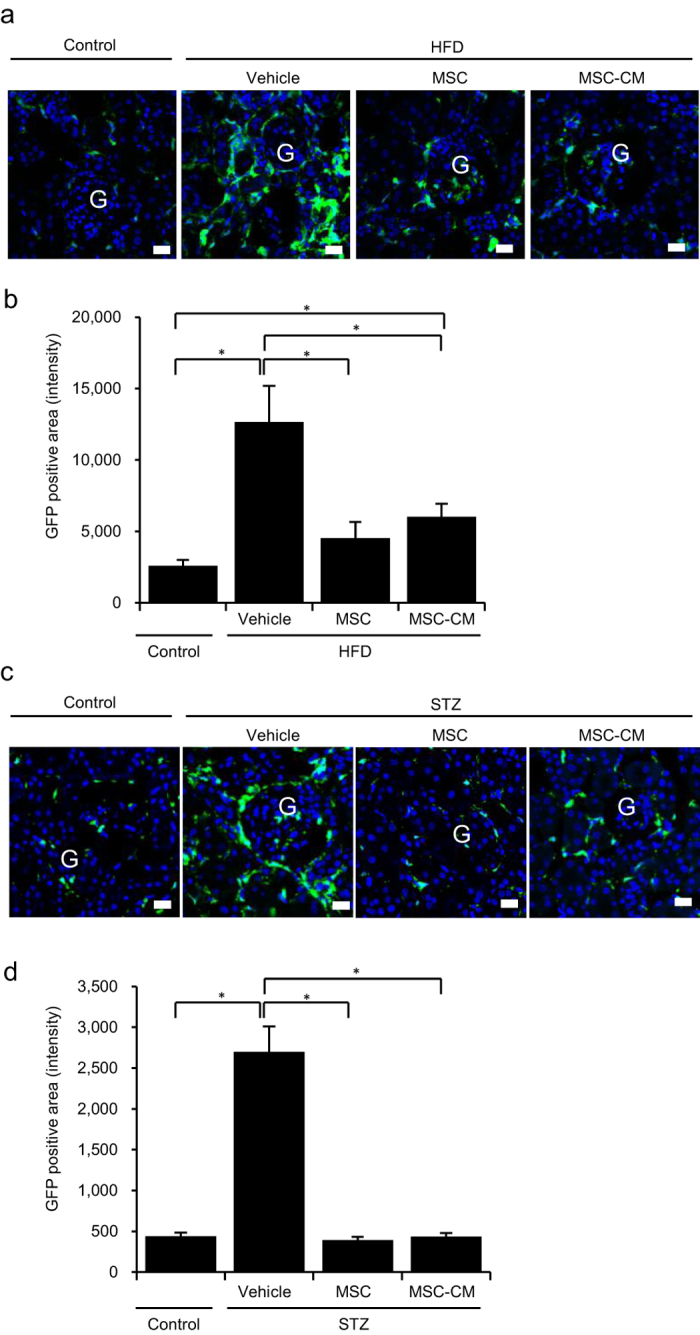
BMDCs infiltrating the kidney of HFD- and STZ-diabetic mice with GFP-chimera. (**a**) Confocal microscopic images of GFP-expressing bone marrow-derived cells (BMDCs) in the kidney of HFD-mice. G: glomerulus. Bar: 20 μm. (**b**) The intensity of the GFP-positive areas in the kidney is quantified. Data are expressed as mean ± SE values of 4–6 animals (mean value of 5 panels per animal). *P < 0.05. (**c**) Confocal microscopic images of GFP-expressing BMDCs in the kidney of STZ-mice. G: glomerulus. Bar: 20 μm. (**d**) The intensity of the GFP-positive areas in the kidney is quantified. Data are expressed as mean ± SE values of 5 animals (mean value of 5 panels per animal). *P < 0.05.

Regions containing GFP-positive-BMDCs (green areas) were significantly larger in HFD-vehicle and STZ-vehicle mice compared with controls (Fig. 3a,c). BMDCs massively infiltrated the interstitial area of the renal tubules and the circumference of Bowman’s capsules. MSC and MSC-CM therapies reversed the increase in the area occupied by infiltrated BMDCs in the kidney of HFD-vehicle and STZ-vehicle mice (Fig. 3b,d).

**Figure 4:**
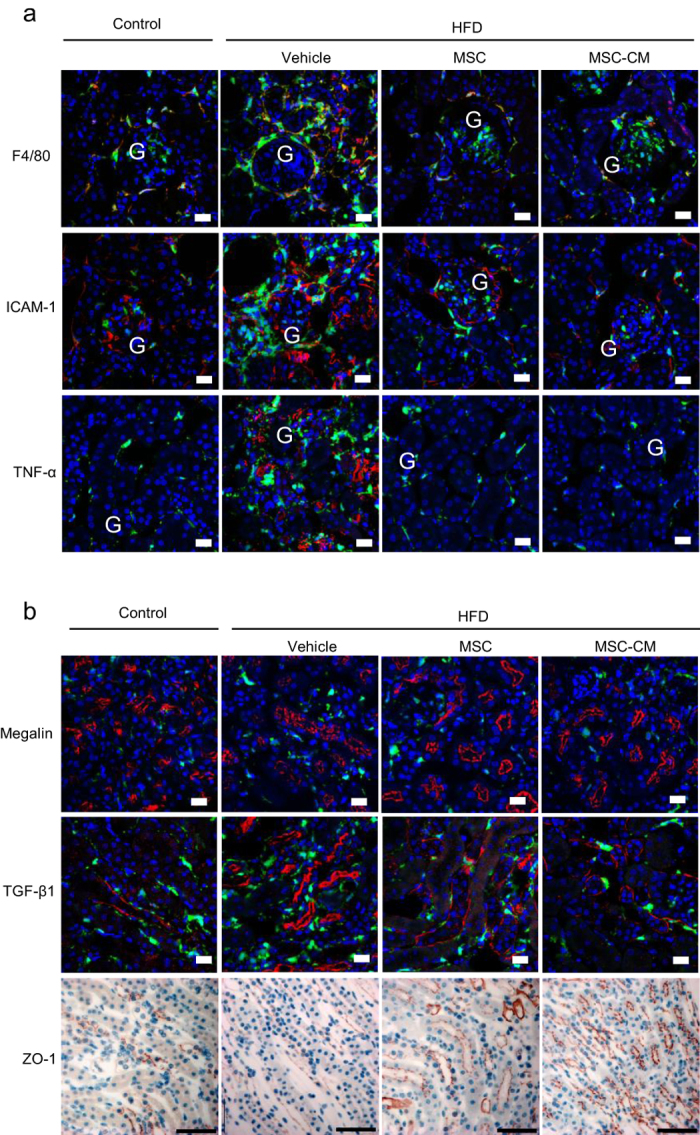
Anti-inflammatory effects of MSC and MSC-CM therapies in the kidney of HFD-diabetic mice. (**a**) Immunofluorescence staining (red) of F4/80, intracellular adhesion molecule-1 (ICAM-1), and tumor necrosis factor alpha (TNF-α) in the kidney. G: glomerulus. Bar: 20 μm. (**b**) Immunofluorescence staining (red) of megalin (upper panel) and transforming growth factor-beta (TGF-β) (middle panel). Bar: 20 μm. Immunochemical staining of zona occludens protein-1 (ZO-1) (lower panel). Bar: 50 μm.

**Figure 5:**
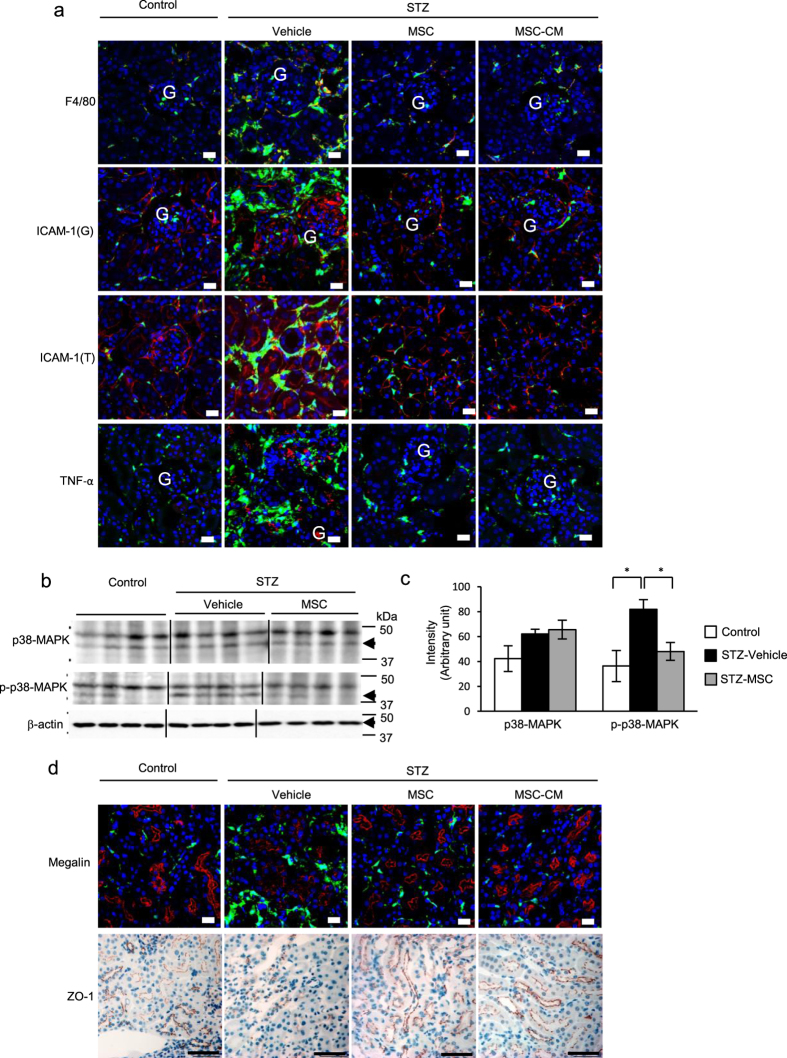
Anti-inflammatory effects of MSC and MSC-CM therapies in the kidney of STZ-diabetic mice. (**a**) Immunofluorescence staining (red) of F4/80, ICAM-1, and TNF-α in the kidney. G: glomerulus; T: tubular epithelial lesion. Bar: 20 μm. (**b**) Immunoblotting of p38-MAPK and phosphorylation of p38-MAPK in renal tissues of STZ-induced diabetic mice. (**c**) Relative amounts of protein are normalized to an internal control, β-actin. Intensity is shown as an arbitrary unit (n = 4, each group). Data are expressed as mean ± SE values *P < 0.05. (**d**) Immunofluorescence staining (red) of megalin (upper panel). Bar: 20 μm. Immunochemical staining of ZO-1 (lower panel). Bar: 50 μm.

We investigated the population of macrophages expressing F4/80 in the BMDCs that infiltrated diabetic renal tissue. In HFD-vehicle and STZ-vehicle mice, infiltration of bone marrow-derived macrophages was substantially increased, but this was returned to normal levels by MSC and MSC-CM therapies (Figs 4a and 5a, upper panels, yellow).

### MSC and MSC-CM therapies down-regulate the expression of adhesion molecules for BMDCs in the kidney

The expression of intracellular adhesion molecule-1 (ICAM-1) was increased in the endothelial cells of peritubular blood vessels and glomerular capillaries in HFD-vehicle and STZ-vehicle mice. Ectopic ICAM-1 expression was observed in the apical site of TECs in STZ-vehicle mice. Its expression was decreased to control levels by MSC and MSC-CM therapies (Figs 4a and 5a, middle panels), which was parallel to the change in BMDC hyperinfiltration of the interstitium of diabetic kidney.

### MSC and MSC-CM therapies suppress proinflammatory cytokine expression in the kidney of HFD- and STZ-diabetic mice

The expression of proinflammatory molecules, such as TNF-α, was up-regulated in renal TECs and interstitial cells of HFD-vehicle and STZ-vehicle mice. Their expression was extremely reduced by MSC and MSC-CM therapies (Figs 4a and 5a, lower panels), which was again parallel to BMDC hyperinfiltration of the interstitium of diabetic kidney.

### MSC therapy regulated inflammatory signaling of renal tissues in STZ-diabetic mice

Phosphorylation of p38 mitogen-activated protein kinase (MAPK) was enhanced in the renal tissues of STZ-vehicle mice, while MSC therapy reversed the activation of p38-MAPK in STZ-diabetic mice (Fig. 5b). The cropped images of immunoblots displayed in the figure and the full-length blots were shown in Supplementary Fig. S3. Relative amount of proteins were shown as an arbitrary unit (Fig. 5c).

### MSC and MSC-CM therapies suppress epithelial damage in proximal tubules in the kidney of HFD- and STZ-diabetic mice

Irregularity and interruption of the localization of megalin, which is normally expressed specifically in the brush border of proximal tubules with a regular sequence, were observed in diabetic kidney. MSC and MSC-CM therapies reversed its expression pattern and increased its intensity in HFD- and STZ-diabetic mice, which was parallel to the morphological changes of TEC damage (Figs 4b and 5d, upper panel).

### MSC and MSC-CM therapies prevent tubular EMT and tight junction protein expression in TECs in the kidney of HFD- and STZ-diabetic mice

TGF-β, which is normally localized in interstitial cells, but induces EMT in TECs and podocytes in DN, was expressed excessively and ectopically in TECs in HFD-vehicle mice. Its expression was remarkably decreased by MSC and MSC-CM therapies (Fig. 4b). Conversely, zona occludens protein-1 (ZO-1), which is expressed in TECs and contributes to the barrier function of epithelial cells in the kidney, was down-regulated in HFD-vehicle and STZ-vehicle mice. MSC and MSC-CM therapies reversed its expression levels and localization in TECs of diabetic mice (Figs 4b and 5d, lower panel).

### Exosomes contained in MSC-CM exert a therapeutic effect on DN *in vivo*

To investigate the major components of MSC-CM that ameliorate DN, we focused on exosomes derived from MSCs as a candidate key factor. Exosomes were isolated from MSC-CM and administrated into the renal subcapsular space of STZ-induced diabetic rats.

**Figure 6:**
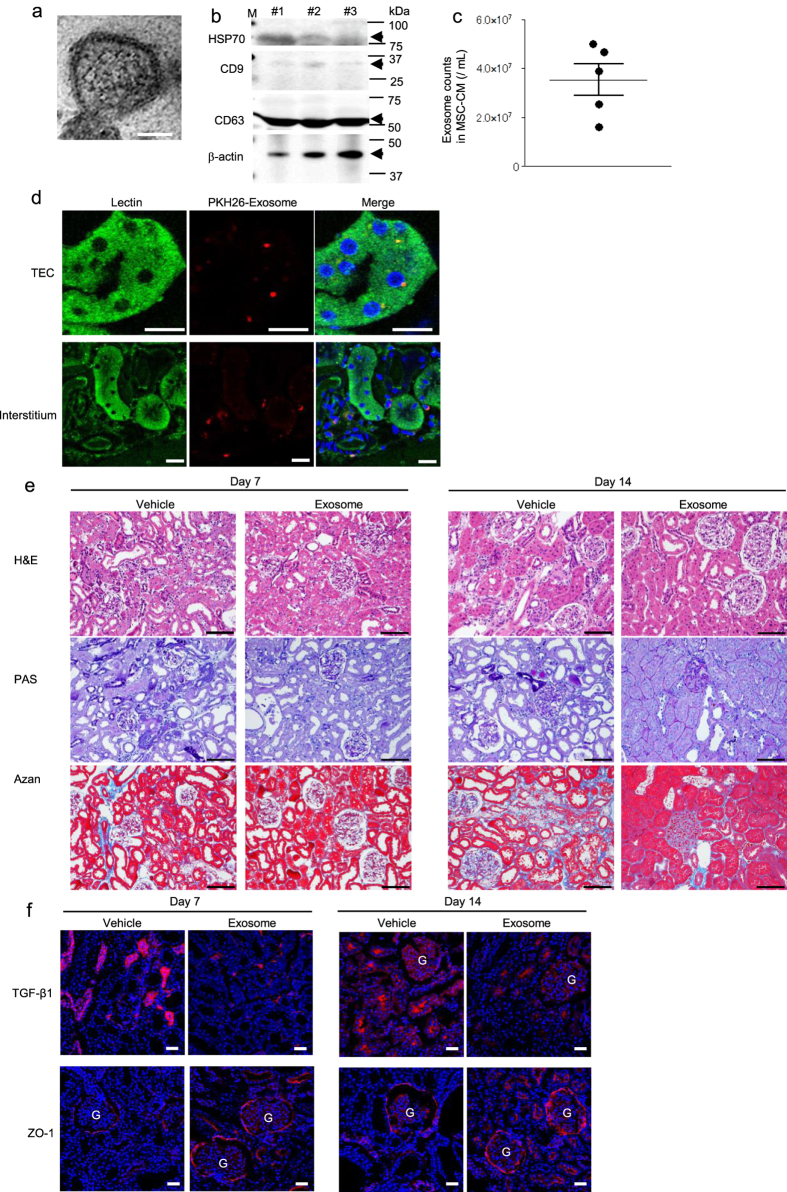
Therapeutic effect of exosomes derived from MSCs for streptozotocin-induced diabetic rats. (**a**) Transmission electron microscopy images of exosomes isolated from MSC-CM. Bar: 20nm. (**b**) Immunoblottingof exosomes using anti-HSP-70, CD9, CD63, and β-actin antibodies. Exosomes isolated from 3 different lots of MSC-CM were shown in the panel. (**c**) The number of exosomes in MSC-CM, which were quantified by CD9-targeting ELISA. Data are expressed as mean ± SE values. (**d**) Immunofluorescence staining of lectin (left panel), distributed PKH26-labeled exosomes (middle panel), and merge image (right panel) of renal tubular epithelial cells (TECs) and interstitial cells in STZ-induced diabetic rats. DAPI was used for counterstaining of nuclei (blue). Bar: 10 μm. (**e**) Histological findings of the renal cortex in H&E-, PAS-, and Azan-stained kidney sections at 7 and 14 days after renal subcapsular administration of exosomes in STZ-diabetic rats. Bar: 100 μm in the panels of H&E, PAS, and Azan staining. (**f**) Immunofluorescence staining of TGF-β1 and ZO-1 in renal tissues at 7 and 14 days after subcapsular administration of exosomes in STZ-diabetic rats. DAPI was used for counterstaining of nuclei (blue). G: glomerulus. Bar: 20 μm.

To examine whether exosomes were specifically purified from MSC-CM, morphological analysis by transmission electron microscopy (TEM) and immunoblotting analysis for typical exosome markers were performed. TEM observations showed microvesicles with a diameter of 40–100 nm having a double membrane that showed the characteristics of exosomes (Fig. 6a). Immunoblotting analysis of pellets obtained from three lots of MSC-CM that were prepared from MSCs of different rats showed they all expressed heat shock protein 70 (HSP70), cluster of differentiation (CD) 9, and CD63 (Fig. 6b). The average count of exosomes in MSC-CM from 5 individual rats was 3.53 × 10^7^ ± 6.42 × 10^6^/mL (Fig. 6c).

Exosomes purified from MSC-CM were administrated into the subcapsular space of unilateral kidney of STZ-induced diabetic rats, and phosphate-buffered saline (PBS) was administered into the subcapsular space of the other kidney as vehicle. PKH26 fluorescence-labeled exosomes were detected in lectin-positive TECs as well as lectin-negative interstitial cells (Fig. 6d).

Histologically, at both 7 and 14 days after therapeutic intervention, the kidney that received exosome injection demonstrated the ameliorated expansion of renal tubules, vacuolation and atrophic change of TECs, and inflammatory cell infiltration, which were observed using H&E staining, degeneration of TECs observed with PAS staining, and increase of the fibrous component of the interstitial space observed with Azan staining compared to the other side with vehicle (Fig. 6e). The expression of TGF-β1 in TECs was suppressed, while ZO-1 expression in Bowman’s capsule and TECs was maintained in the injected side of the kidney (Fig. 6f).

### Exosomes suppress apoptosis and degeneration of TECs in primary renal cell (PRC) culture of STZ-induced diabetic rats

**Figure 7:**
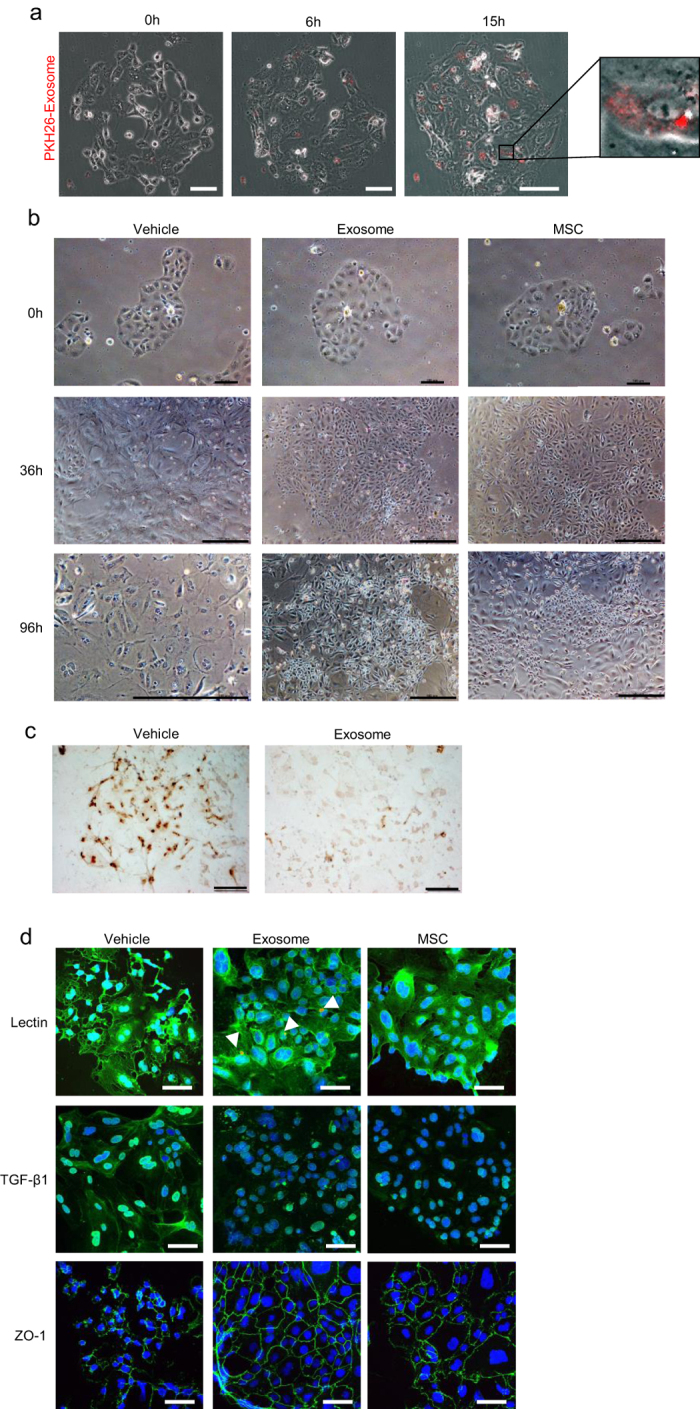
Anti-apoptotic and anti-degenerative effects of exosomes and co-cultured MSCs on tubular epithelial cells (TECs) in primary renal cell culture of streptozotocin-induced diabetic rats. (**a**) Time-lapse images of the incorporation of fluorescence-labeled exosomes on primary renal cell (PRC) culture. The images were obtained immediately (0 h), 6 h, and 15 h after adding exosomes to the culture medium. Bar: 100 μm. (**b**) Phase contrast observations of PRCs cultured without (left panel) or with exosomes (middle panel) and co-cultured with MSCs using a transwell (right panel). The images were obtained immediately (0 h), 36 h, and 96 h after adding exosomes to the culture medium or starting co-culture with MSCs. Bar: 100 μm in 0 h; 500 μm in 36 h and 96 h. (**c**) Apoptotic images of PRCs due to the presence or absence of exosomes and cultured for 96 h. Bar: 100 μm. (**d**) Immunofluorescence staining of lectin, TGF-β1, and ZO-1 in PRCs cultured without (left panel) or with exosomes (middle panel) and co-cultured with MSCs using a transwell (right panel) for 96 h. DAPI was used for counterstaining of nuclei (blue). Bar: 50 μm.

In the initial phase of PRC culture, TECs with strong proliferative ability and a few interstitial cells were observed. PKH26 fluorescence-labeled exosomes that were added to the medium were captured by TECs and the number of captured exosomes increased in a time-dependent manner (Fig. 7a). Colonies of TECs isolated from STZ-induced diabetic rats were skewed after 36 h of incubation, and the cells were degenerated and had entered apoptosis after 96 h of incubation (Fig. 7b, left panels, Fig 7c, left panel). Conversely, TECs to which exosomes were added to their culture medium or indirectly co-cultured with MSCs were protected from these degenerative changes and continued to expand colonies in which the TECs were arranged in a compact manner (Fig. 7b, middle and right panels, Fig. 7c, right panel).

The expression of lectin was reduced along with deformation of the cytoplasm in TECs after 96 h incubation in vehicle, while its expression was maintained in TECs cultured with exosomes or MSCs. PKH26 fluorescence-labeled exosomes were detected (Fig. 7d, upper middle panel). TGF-β1 was highly expressed in TECs that were transformed into a spindle-shape in vehicle, while these changes were suppressed by culturing with exosomes or MSCs (Fig. 7d, middle panels). ZO-1 expression was reduced in vehicle, while it was firmly maintained in TECs cultured with exosomes or MSCs (Fig. 7d, lower panels).

## Discussion

In the present study, we investigated whether MSCs could ameliorate DN in HFD- and STZ-induced diabetic models by an anti-inflammatory mechanism through inhibition of the hyperinfiltration of BMDCs, an anti-fibrotic action, and protection of TECs from degeneration and EMT. We found that exosomes derived from MSCs might be partially involved in these functions.

The curative effects of the intravenous administration of MSCs and MSC-CM were almost similar. As only very small numbers of donor MSCs were observed in the kidney, the therapeutic effects of MSCs on renal damage appeared attributable to various paracrine factors. We have previously reported that the major components of cytokines in MSC-CM were vascular endothelial growth factor (VEGF) and monocyte chemotactic protein 1^34^. In the present study, we found that MSC-CM contains abundant quantities of exosomes, which are known to contain various factors contributing to anti-inflammation, repair, and regeneration of renal tissues^32^. Exosomes are vesicles with a lipid bilayer and diameter of 40–100 nm, which contain mRNAs, DNA, and miRNAs and mediate the transmission of information between cells^28^. We demonstrated that exosomes obtained from multiple lots of MSCs expressed typical markers such as HSP70, CD9, and CD63 by immunoblotting analysis. The number of exosomes was quantified using an enzyme-linked immunosorbent assay (ELISA) and then approximately 5.3 × 10^7^ exosomes were administered per kidney, which was the number of exosomes isolated from the culture supernatant of MSCs of the same number which was administered systemically. Although the number of exosomes injected locally seems to be higher than that derived from systemically administered MSCs, we obtained similar effects in injured kidney between systemically administered MSC-CM and locally injected exosomes. Therefore, exosomes contained in MSC-CM were considered to be a key factor for the therapeutic effects of MSCs.

Since paracrine factors which MSC secreted, including exosomes, shows great variety depending on the individual difference, culture conditions of MSCs, for example, passage number of cells, the dimension of the culture environment (two-dimension or three-dimension) and the culture medium, it is difficult to standardize the contents of MSC-CM and its efficacy for renal damages. So we ensured the identity of the culture supernatant of MSC including exosomes by culturing in exactly the same protocol to evaluate its contents and therapeutic effects on diabetic nephropathy.

MSC and MSC-CM therapies completely reversed the significant increase in BMDC infiltration into the kidney of HFD- and STZ-induced diabetic mice. Approximately 50% of the infiltrated BMDCs were identified as F4/80-positive macrophages, which induce the production of pro-inflammatory cytokines, such as TNF-α. ICAM-1 expression in peritubular capillaries was enhanced in HFD- and STZ-diabetic mice, and was suppressed by the MSC and MSC-CM therapies. ICAM-1 signals are known to be activated in the tubular epithelia and peritubular capillaries in association with advanced glycation end product (AGE) deposition^35^, which enhances the recruitment of inflammatory cells from vessels. Interestingly, ICAM-1 was also expressed ectopically in TECs in STZ-diabetic mice in the present study. This expression might contribute to the excessive infiltration of BMDCs and the disorder of TECs.

MSC and MSC-CM therapies suppressed tubular interstitial fibrosis, reversed the enhanced and ectopic expression of TGF-β, and prevented ZO-1 degradation in TECs. Fibrotic changes were observed in the tubulointerstitium of HFD- and STZ-induced diabetic mice. Furthermore, we observed the strong expression and abnormal distribution of TGF-β, which was localized not in the basal membrane but in TECs ectopically, in HFD-diabetic mice, as well as down-regulation of the tight junction protein ZO-1 in TECs in HFD- and STZ-diabetic mice. Previous studies have focused on EMT of proximal tubular cells and podocytes as a major source of myofibroblasts and critical for the induction of fibrosis in DN^11^,^12^,^36^. TGF-β, which is upregulated in DN by high glucose, AGEs, and albumin overload, is a key regulator of EMT induction by dissolution of tight junction proteins in renal tubular cells^37^,^38^. In the present study, we showed the inhibitory effects of MSC and MSC-CM therapies on the overexpression of TGF-β in TECs and fibrosis in peritubular epithelial lesions in DN.

Excessive dilatation of tubules and degeneration of TECs were suppressed by the MSC and MSC-CM therapies. Vacuolization, atrophic changes, accumulation of denatured substances, which were strongly positive in PAS staining, and a decrease of megalin expression were observed in TECs in the kidney of HFD- and STZ-diabetic mice. The severe degeneration of TECs was reduced and megalin expression in proximal TECs was recovered by the MSC and MSC-CM therapies. We showed that exosomes derived from MSC-CM could protect TECs from apoptosis and maintained tight junction structural proteins that are responsible for the barrier function of tubules. In acute renal injury, exosomes derived from MSCs demonstrate protective effects via the transportation of a specific subset of cellular mRNAs, which are associated with the mesenchymal phenotype and with the control of transcription, proliferation, and immunoregulation^39^,^40^. Bruno *et al*. reported that exosomes activated a proliferative program of TECs and inhibited apoptosis via inducing the synthesis of hepatocyte growth factor and macrophage-stimulating protein by the horizontal transfer of mRNAs packed in exosomes^40^. Tomasoni *et al*. reported that the repair of cisplatin-damaged proximal tubular cells by MSCs was induced by the combined trophic effect of insulin-like growth factor 1 (IGF-1) released by bone marrow-MSCs and the transfer of the mRNA of the corresponding IGF-1 receptor via exosomes^31^. In the present study, it was considered that mRNAs and/or miRNAs carried by exosomes played a role in the protection and regeneration of TECs.

MSC and MSC-CM therapies reduced the expansion of glomerular mesangium substrate in HFD-diabetic mice. Down-regulation of VEGF has been reported to contribute the abnormal mesangial expansion. Since MSC-CM includes abundant VEGF, as we showed previously^34^,^41^, MSC and MSC-CM therapies may contribute to the improvement of the glomerular disorder in this manner.

Administration of MSC and MSC-CM reduced urine albumin secretion despite unattenuated hyperglycemia in diabetic mice. Diabetic nephropathy is caused mostly by cellular metabolic disorders due to hyperglycemia, which induce functional alterations in renal microvasculature, glomeruli and tubular epithelium and promotes urinary albumin excretions. On the other hands, other factors such as oxidative stress, inflammation, interstitial fibrosis, excessive production of extracellular matrix (ECM) through activating TGF-β signaling have been reported to explain the mechanism of histopathological abnormalities in DN. Wu H reported that inhibition of c-Src/p38-MAPK pathway ameliorated tubular epithelial cell apoptosis in db/db mice and renal epithelium *in vitro* which were cultured in high glucose conditions. This result indicated that tubular epithelial damages were suppressed at least in part by regulating p38 MAPK pathway even under a hyperglycemia^42^. Li H reported that ECM synthesis, which induce renal and vascular scarring, were dependent on Smad/TGF-β signaling under high glucose conditions, and it was blocked by neutralizing TGF-β antibody^43^. In this study, we demonstrated that activation of p38-MAPK and TGF-β expression was suppressed by MSC therapies in hyperglycemic mice. These results suggested that MSC therapies may improve renal disorder in hyperglycemic DN by suppressing inflammatory signaling and fibrosis.

**Figure 8:**
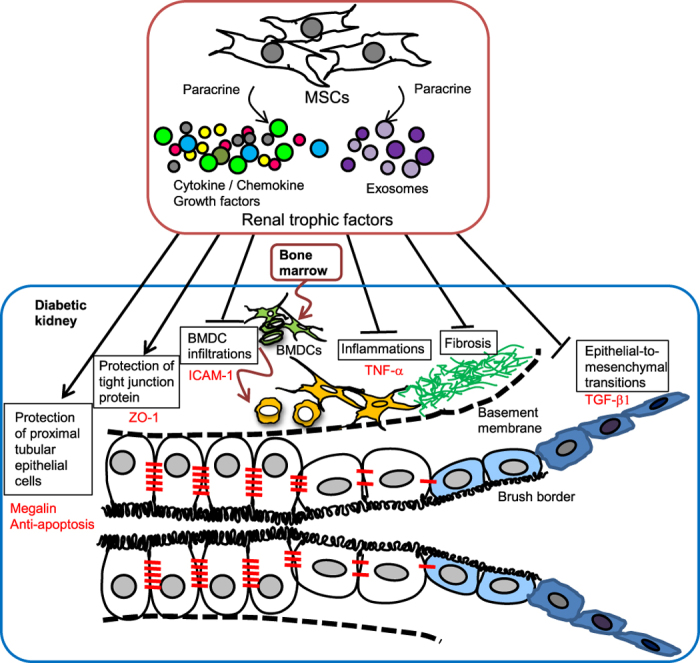
Proposed mechanisms for the therapeutic effects of MSCs for DN. Site of action of MSC therapy for DN.

In conclusion, the site of action of MSC therapy for DN presented in this study is shown in Fig. 8. We demonstrated that MSC therapy is a promising tool for repairing DN not only in STZ-induced insulin-deficient T1D mice but also HFD-induced T2D mice. The effects of MSC therapy might be due to MSC-derived trophic factors, including exosomes. MSC and MSC-CM therapies suppressed abnormal BMDC infiltration of the kidney and interstitial fibrosis with excessive expression of proinflammatory cytokines. These therapies reversed the serious damage to TECs and suppressed EMT. Furthermore, glomerular alterations were inhibited in HFD-diabetic mice. As the pathophysiological condition of DN is induced by various disorders, an MSC therapy capable of acting pleiotropically is very useful with its comprehensive effectiveness in treating DN, and these results may provide a gateway for new therapeutics.

## Methods

### Animal models of diabetes and bone marrow transplantation

All methods were carried out in accordance with the approved guidelines. Eight-week-old male C57BL/6J mice and C57BL/6-Tg (CAG-EGFP) (GFP-transgenic; GFP-Tg) mice were purchased from Japan SLC (Shizuoka, Japan). GFP-bone marrow chimeric mice were produced by lethal irradiation (9 Gy) and systemic injection with 4.0–6.0 × 10^6^ bone marrow cells isolated from GFP-Tg mice. At 4 weeks after bone marrow transplantation, diabetes was induced via an HFD containing 60% lard (High-Fat Diet 32; Clea Japan, Inc., Tokyo, Japan) or by a single intraperitoneal injection of STZ (150 mg/kg; Wako, Osaka, Japan) dissolved in citrate buffer (pH 4.5). Control mice were given a normal diet or treated with buffer intraperitoneally. After 28 weeks of receiving the HFD, the mice were administered 1.0 × 10^4^ MSCs/g body weight 4 times (HFD-MSC) every 2 weeks, with the controls receiving buffer (HFD-vehicle) (Fig. 1a). At 4 weeks after STZ injection, the mice were administered 1.0 × 10^4^ MSCs/g body weight 2 times (STZ-MSC) every 4 weeks, with the controls receiving buffer (STZ-vehicle) (Fig. 2a). All experimental protocols and studies were approved by the animal experiment committee of Sapporo Medical University (Sapporo, Japan).

### Biochemical tests for albuminuria and glucose levels

Urine samples were collected from HFD- and STZ-diabetic mice. The mice were housed in metabolic cages and whole urine was collected for 3 h. Analysis of albumin and creatinine levels in the urine was delegated to SRL, Inc. (Tokyo, Japan). Albumin levels were measured by an immune-turbidimetric method, and creatinine levels were measured by an enzymatic method. Urinary albumin excretion was normalized to urinary creatinine excretion. Blood samples were collected after 12 h fasting from HFD-diabetic mice and occasionally from STZ-diabetic mice. Successful establishment of the diabetes model was confirmed via blood glucose monitoring using a glucometer system (Nipro Carefast Meter; NIPRO Corporation, Osaka, Japan).

### Isolation, culture, and characterization of rat MSCs

Bone marrow fluids were collected from bone marrow of 8-week-old Lewis rats (Charles River Laboratories Japan, Inc., Yokohama, Japan) and SD-Tg (CAG-EGFP) (Sankyo Labo Service Corporation, Inc., Tokyo, Japan). Rat MSCs were harvested by adherent cultures of bone marrow cells as described previously^44^. Characterization of rat MSCs was determined by fluorescence-activated cell sorting (Calibur; BD Bioscience, San Jose, CA) using the rat surface antigen-specific antibodies CD90, CD44, CD45, CD43, CD31, and CD11b (Immunotech-Beckman Coulter, Marseille, France). The immunophenotype of rat MSCs was determined (Supplementary Fig. S1).

### Preparation of MSC-CM from rat MSCs

MSCs isolated from Lewis rats (passage 3) were seeded in 150-cm^2^ culture dishes, and medium was changed to serum-free medium when confluence was reached. The cells were then cultured for 24 h and supernatant was collected and concentrated (final concentration: 1 mg/mL) by ultrafiltration using centrifugal filter units with a 10 kDa cut-off (Ultracel-10K; Millipore, Billerica, MA) to produce MSC-CM, following the manufacturer’s instructions.

### Isolation and characterization of exosomes derived from rat MSCs

MSCs were cultured and expanded to passage 3 as described above. To isolate exosomes, MSCs were cultured for 48 h with medium containing exosome-depleted fetal bovine serum (FBS) (System Biosciences, Inc., Mountain View, CA). After the culture media were collected, exosomes were isolated using Total Exosome Isolation Reagent (Invitrogen, Carlsbad, CA) according to the manufacturer’s instructions. The isolated exosomes were characterized morphologically by TEM observation and immunoblotting for the expression of HSP70, CD9, and CD63, as described in the Supplementary Methods in detail. The number of exosomes was analyzed using a CD9 ELISA (System Biosciences, Inc., Mountain View, CA) according to the manufacturer’s instructions.

### Fluorescence labeling of exosomes

Exosomes were stained with the red fluorescence dye PKH26 (Sigma-Aldrich, St. Louis, MO) as described previously^45^. Briefly, the exosomes were suspended in diluent C (Sigma-Aldrich, St. Louis, MO) and incubated with PKH26 at room temperature for 5 min. The reaction was stopped by the addition of FBS and complete medium. The PKH26-labeled exosomes were precipitated again by ultracentrifugation at 100,000 × *g* at 4 °C for 1 h using an ultracentrifuge (Hitachi, Tokyo, Japan).

### Intravenous administration of MSCs and MSC-CM

Mice were administered MSCs (1.0 × 10^4^ MSCs/g body weight per animal suspended in 200 μL PBS) or MSC-CM (2 mg/kg/day) via the tail vein after the induction of diabetes (Figs 1a and 2a). MSC-CM was also administered to diabetic mice daily for 8 weeks (Figs 1c and 2c). Vehicle mice were instead administered PBS via the tail vein and serum-free medium concentrated following the same methods as the conditioned medium used for MSC-CM therapy.

### Renal subcapsular administration of exosomes derived from rat MSCs

To evaluate whether exosomes derived from MSCs can ameliorate the histological damage of renal tissue in STZ-induced diabetic rats, 5.3 × 10^7^ exosomes, which were evaluated by ELISA, were suspended in 200 μL PBS and injected into the subcapsular space of a unilateral kidney. The same amount of PBS was administered as a vehicle in the subcapsular space of the opposite kidney. Kidney tissues were obtained at 7 and 14 days after the administration of exosomes to evaluate histological findings.

### Primary culture of renal tissues

To evaluate the direct effect of exosomes and indirect effects of MSCs on renal TECs from STZ-induced diabetic rats, PRCs were prepared *in* vitro according to a protocol that was reported previously^46^,^47^. Briefly, kidneys were removed from STZ-induced diabetic rats and minced with 1 mg/mL collagenase (Sigma-Aldrich, St. Louis, MO) diluted in PBS for 30 min at 37 °C. Dissolved tissue was filtered with a 100 μm pore cell strainer and centrifuged for 5 min at 400 × *g*. The cell pellet was treated with RBC lysis buffer (QIAGEN, Hilden, Germany). Then, 5.0 × 10^5^ cells were plated onto 4-well chamber slides or 1.0 × 10^6^ cells were placed in a 12-well multiwell plate in F-12/DMEM with 10% FBS (Sigma-Aldrich), 100 U/mL penicillin, and 100 μg/mL streptomycin (GIBCO, Palo Alto, CA), Insulin-Transferrin-Selenium-X (Invitrogen, Carlsbad, CA), prostaglandin E1 (7.1 × 10^−8^ M; Sigma-Aldrich, St. Louis, MO), triiodothyronine (2.0 × 10^−9^ M; Sigma-Aldrich, St. Louis, MO), and dexamethasone (5.09 × 10^−8^ M; Sigma-Aldrich, St. Louis, MO) as reported previously^29^. The medium was changed at 48 h after initial plating. After 24 h, exosomes were added to the cells or they were co-cultured with MSCs using a transwell and incubated for 96 h. Incorporation of fluorescence-labeled exosomes into PRCs (mainly TECs) was observed at 0 h, 6 h, and 15 h after exposure to exosomes or MSCs on phase contrast and fluorescence microscopy (Axio Observer Z1; Carl Zeiss, Oberkochen, Germany) at 37 °C and 5% CO_2_ using a stage top incubator (TOKAI HIT, Fujinomiya, Japan) and time-lapse observations were carried out. At 96 h after exposure to exosomes or MSCs, PRCs were fixed with 4% paraformaldehyde, and terminal deoxynucleotidyl transferase-mediated dUTP nick-end labeling (TUNEL) reactions were conducted to evaluate the state of apoptosis of TECs using the DeadEnd Colorimetric TUNEL system (Promega, Madison, WI). Lectin, TGF-β1, and ZO-1 were stained using the respective primary and secondary antibodies listed in Supplementary Tables S1 and S2. Images were taken under a confocal laser scanning microscopy (LSM 510; Carl Zeiss, Oberkochen, Germany).

### Optical microscope observations of renal tissues

Paraffin-embedded kidney tissues were cut into thin sections, stained with H&E (Wako, Osaka, Japan), PAS, and Azan (Muto Pure Chemicals, Tokyo, Japan), and viewed with a light microscope (NIS element BR 3.0; Nikon, Tokyo, Japan). The extent of renal tissue disorders were evaluated focusing on glomeruli, tubular epithelium, and interstitial changes according to previous reports^18^,^48^. Briefly, glomerular damage was expressed as the percentage of glomeruli presenting mesangial expansion or glomerulosclerosis. Impairment of tubular epithelium was graded from 0 to 4 according to the presence of tubular dilatation, protein cylinders, and atrophic changes (0, no change; 1, change of 25% or less; 2, change from 25% to 50%; 3, change from 50% to 75%; 4, more than 75% change).

### Detection of donor MSCs

HFD- and STZ-induced diabetic mice without bone marrow transplantation were administered MSCs that were marked with fluorescent substances. Mice that had received labeled MSCs were killed at 1, 2, or 4 weeks after MSC injection. Liver, bone, kidney, lung, and spleen were obtained and digested into single cells to detect fluorescence-labeled MSCs in each organ. The number of fluorescent MSCs distributed in each organ was analyzed by flow cytometry and immunofluorescence observations by confocal laser scanning microscopy (LSM 510; Carl Zeiss, Oberkochen, Germany).

### Detection of fluorescence-labeled exosomes *in vivo*

STZ-induced diabetic rats were administered exosomes that were marked with fluorescent PKH26. Rats that had received labeled exosomes were sacrificed at 7 and 14 days after exosome administration. Kidney was obtained and the distribution of fluorescence labeled-exosomes was analyzed by immunofluorescence observations using a confocal laser scanning microscope (LSM 510; Carl Zeiss, Oberkochen, Germany). An anti-lectin antibody was used to stain the cytoplasm of TECs.

### Quantitative analysis of GFP-positive areas

Kidney samples were immersed in 4% paraformaldehyde, cryosectioned, and observed with confocal laser scanning microscopy. Ten randomly selected visual fields were observed at × 100 magnification using the NIS element BR 3.0 image analysis system to assess the mean percentage of area occupied by GFP-positive cells in the kidney of each animal.

### Immunofluorescence staining

Immunofluorescence staining of target factors was performed. Kidney samples were immersed in 4% paraformaldehyde, cryosectioned, and incubated with primary and secondary antibodies (Supplementary Tables S1 and S2). Nuclei were stained with DAPI and observed by confocal laser scanning microscopy (LSM 510; Carl Zeiss, Oberkochen, Germany).

### Immunohistochemistry

Immunochemical staining was performed using an avidin-biotin-based system. Kidney samples were immersed in 4% paraformaldehyde and paraffin-embedded sections were incubated with primary antibodies (Supplementary Table S1), biotinylated secondary antibody (Dako, Glostrup, Denmark), and streptavidin-conjugated horseradish peroxidase (Dako, Glostrup, Denmark). The signal was developed with diaminobenzidine (Sigma-Aldrich, St. Louis, MO). Nuclei were stained with hematoxylin and observed by light microscopy.

### Immunoblotting of renal tissues

Inflammatory signaling of renal tissues was analyzed by immunoblotting of p38-MAPK and phosphorylation of p38-MAPK as described in the Supplementary Methods in detail.

### Statistical analysis

Data are expressed as mean ± standard error (SE) values. Analysis of variance was employed for multiple comparisons. Two-way repeated measures (mixed between-within subjects) analysis of variance followed by Bonferroni’s test was used for serial assessments. Statistical analysis was performed using GraphPad Prism 6.0 (GraphPad Software, Inc., San Diego, CA). Differences were considered significant at *P* < 0.05 for all two-tailed tests.

## Additional Information

**How to cite this article**: Nagaishi, K. *et al*. Mesenchymal stem cell therapy ameliorates diabetic nephropathy via the paracrine effect of renal trophic factors including exosomes. *Sci. Rep.* **6**, 34842; doi: 10.1038/srep34842 (2016).

## Supplementary Material

Supplementary Information

## Figures and Tables

**Table 1:** Blood glucose levels in diabetic mice.

Period after the initial administration of MSCs or MSC-CM		
Group	0 w	8 w
Control	86.8 ± 5.7	91.3 ± 5.4
HFD-Vehicle	166.0 ± 16.7*	188.8 ± 14.1*
HFD-MSC	188.6 ± 11.8*	143.0 ± 9.0*^#^

Control	92.4 ± 3.0	101.8 ± 5.4
HFD-Vehicle	212.2 ± 3.3*	178.0 ± 15.2*
HFD-MSC-CM	183.9 ± 10.0*	152.3 ± 5.2*

Control	141.0 ± 4.2	192.0 ± 5.8
STZ-Vehicle	486.7 ± 35.3**	565.0 ± 24.7**
STZ-MSC	531.7 ± 11.0**	503.2 ± 24.9**

Control	197.8 ± 7.2	188.0 ± 3.8
STZ-Vehicle	535.8 ± 37.9**	554.2 ± 27.4**
STZ-MSC-CM	541.7 ± 20.5**	519.8 ± 30.0**

Blood glucose levels in high-fat diet (HFD)- and streptozotocin (STZ)-diabetic mice at pre-treatment and after 8 weeks of initial treatment with the MSC and MSC-CM therapies. Unit: mg/dL. Data are expressed as mean ± SE values of 5–7 animals. *P < 0.05 HFD-vehicle or HFD-MSC or HFD-MSC-CM vs. each control; **P < 0.05 STZ-vehicle or STZ-MSC or STZ-MSC-CM vs. each control; ^#^*P* < 0.05 HFD-MSC vs. HFD-vehicle.
