# Supplementary material for: Mesenchymal stem cell therapy ameliorates diabetic nephropathy via the paracrine effect of renal trophic factors including exosomes
Source: Sci Rep. 2016 Oct 10;6:34842. doi: 10.1038/srep34842 (PMC5056395; doi:10.1038/srep34842)
Supplement: Supplementary Information [file srep34842-s1.pdf]

SUPPLEMENTARY INFORMATION

1. Supplementary Results

2. Supplementary Methods

3. Supplementary References

4. Supplementary Table and Figure legends

5. Supplementary Table

6. Supplementary Figure

Title:

Mesenchymal stem cell therapy ameliorates diabetic nephropathy via the paracrine effect of renal trophic factors including exosomes

Authors:

Kanna Nagaishi, Yuka Mizue, Takako Chikenji, Miho Otani, Masako Nakano, Naoto Konari, Mineko Fujimiya

SUPPLEMENTARY RESULTS

Immunophenotype of rat MSCs

MSCs expressed CD90 as a cell surface antigen, while CD44, CD45, CD43, CD31, and CD11b were not expressed, as described previously for the characteristics of rat MSCs (Supplementary Fig. S1).

Distribution of administered MSCs in diabetic mice

Very few PKH26-positive MSCs were detected in the tubular epithelial area of the kidney in HFD-MSC mice at 7 days after the administration of MSCs (Supplementary Fig. S2a). GFP-positive MSCs were almost undetected in the kidney of STZ-MSC mice, while small numbers of administered MSCs were detected in the spleen of STZ-MSC mice (Supplementary Fig. S2b).

SUPPLEMENTARY METHODS

Isolation, culture, and characterization of rat bone marrow-derived MSCs

Bone marrow fluids were harvested from the bone marrow of 8-week-old Lewis rats (Charles River Laboratories Japan, Inc., Yokohama, Japan) and 8-week-old SD-Tg (CAG-EGFP) (Sankyo Labo Service Corporation, Inc., Tokyo, Japan). Bone marrow MSCs were cultured as described previously[1]. Briefly, bone marrow cells were

harvested from femurs and tibias by flushing whole bone marrow with complete α-modified Eagle's medium (α-MEM; Invitrogen, Carlsbad, CA) containing 15% fetal bovine serum and 1% penicillin-streptomycin. Single cell suspensions were filtered through a 70-μm nylon filter (Becton Dickinson, Franklin Lakes, NJ) and plated in 75-cm^2 flasks. The cells were grown in complete α-MEM at 37°C and 5% CO_2. After 72 h, the medium was replaced with fresh medium, and adherent cells grown to 80% confluency to obtain samples were defined as passage 0. Cells in passage 3 were used for experiments.

Detection of donor MSCs

HFD- and STZ-induced diabetic mice without bone marrow transplantation were administered MSCs isolated from SD-Tg (CAG-EGFP) (Sankyo Labo Service Corporation, Inc., Tokyo, Japan) rats or MSCs isolated from Lewis rats (Charles River Laboratories Japan, Inc., Yokohama, Japan) that were labeled with a PKH26 Red Fluorescent Cell Linker Kit (Sigma-Aldrich, St. Louis, MO). Mice administered MSCs derived from CAG-EGFP rats were sacrificed at 1, 2, or 4 weeks after MSC injection, and lung, liver, kidney, spleen, and bone were obtained. Each tissue was digested into single cells with collagenase (Sigma-Aldrich, St. Louis, MO) and the number of

GFP-positive MSCs distributed in each organ was analyzed by flow cytometry. The organs obtained from mice, which were administered PKH26-labeled MSCs, were also immersed in 4% paraformaldehyde and bone was decalcified with 0.5 M EDTA (Wako, Osaka, Japan) for 2 days. Frozen sections of each organ were stained with DAPI (Dojindo Laboratories, Kumamoto, Japan) at 0.1 mg/mL. The distribution of MSCs expressing red fluorescence in each organ was observed by confocal laser scanning microscopy (LSM 510; Carl Zeiss, Oberkochen, Germany). The ratio of MSCs distributed in each organ was determined by counting PKH26-positive cells in 10 randomly selected visual fields at ×100 magnification per mouse (n = 3–5) and compensated for by the number of MSCs given to each mouse.

Transmission electron microscopic observation of exosomes

Exosome pellets isolated from MSC-CM were fixed for 24 h with 2.5% glutaraldehyde (Wako Pure Chemical Industries, Ltd., Osaka, Japan). The samples were washed with PBS, fixed with 1% osmium tetroxide solution (TAAB Laboratories Equipment Ltd., Aldermaston, UK), and dehydrated with ethanol (Wako Pure Chemical Industries, Ltd., Osaka, Japan). After soaking the samples in propylene oxide (KANTO KAGAKU, Tokyo, Japan), they were embedded with an epoxy resin (TAAB Laboratories Equipment Ltd.) and polymerized with heating. Ultrathin sections of the

samples were prepared using an ultra-microtome (MT-X; RMC Boeckeler Instruments, Inc., Tucson, AZ). The sections were observed with a transmission electron microscope (H7650; Hitachi High-Technologies Corporation, Tokyo, Japan) after electron staining was performed.

Immunoblotting

The molecular content of kidney and exosome was analyzed by immunoblotting. Kidney tissues and exosome pellets isolated from MSC-CM were lysed in Radio Immuno Precipitation Assay buffer that included 20 mM Tris-HCl, pH 7.4, 150 mM sodium chloride, 1 mM EDTA (Sigma-Aldrich, St. Louis, MO), pH 8.0, 0.1% (w/v) sodium dodecyl sulfate, 0.1% sodium deoxycholate, 1% Triton X-100, and 1 tablet of complete Mini™ (Roche Diagnostics, Mannheim, Germany) and Phos STOP (Roche Diagnostics). Five to 40 μg of each lysate, as determined using a Bicinchoninic Acid (BCA) Protein Assay Kit (Thermo Fisher Scientific, Waltham, MA), were resolved on a 12% denaturing polyacrylamide gel and transferred to a polyvinylidene difluoride membrane. After blocking with 5% nonfat dry milk in Tris-buffered saline with Tween 20, the membrane was incubated with primary antibodies (Supplemental Table 1). It was then incubated with horseradish peroxidase-conjugated secondary antibodies (Supplemental Table 2). Immuno-reactivity was developed using an enhanced

chemiluminescence kit (Amersham Biosciences, Piscataway, NJ).

SUPPLEMENTARY REFERENCE

1 Javazon, E. H., Colter, D. C., Schwarz, E. J. & Prockop, D. J. Rat marrow stromal cells are more sensitive to plating density and expand more rapidly from single-cell-derived colonies than human marrow stromal cells. *Stem cells* **19**, 219-225, doi:10.1634/stemcells.19-3-219 (2001).

SUPPLEMENTARY TABLE and FIGURE LEGENDS

Supplementary Table S1. Primary antibodies

The primary antibodies used for immunofluorescence and immunohistochemistry are listed according to species, reaction, and manufacturer. Abbreviations: Abs (P/M), antibodies (polyclonal/monoclonal); Gt, goat; Hu, human; Ms, mouse; Rab, rabbit; Rt, rat; Chk, chicken; GP, Guinea pig; Pg, pig; Dg, dog; B, bovine; Sh, sheep; Ct, cat; Cp, carp; Mk, monkey; Dm, D. melanogaster; Sc, S. cerevisiae.

Supplementary Table S2. Secondary antibodies

The secondary antibodies used for immunofluorescence are listed according to species, conjugate, and manufacturer. Abbreviations: Abs, antibodies; Gt, goat; Dnk, donkey.

Supplementary Figure S1. Immunophenotype of rat MSCs

Flow cytometry analysis of the expression of cell surface markers related to rat MSCs. The green line shows the population stained with the target antibody. The purple line shows the population stained with the isotype control antibody.

Supplementary Figure S2. Distribution of rat MSCs in diabetic mice

(a) Distribution of MSCs marked with PKH26 in the kidney of HFD mice at 1 week after the initial MSC injection is shown in the panel. Bar: 20 μm.

(b) Distribution of GFP-positive MSCs in the kidney and spleen of STZ mice at 1 week after the initial MSC injection is shown. Flow cytometry analysis of GFP-positive cells is shown in the left panels. The ratio of GFP-positive MSCs in total cells isolated from kidney and spleen is shown in the right panel. Data are expressed as mean ± SE values of 4 animals.

Supplementary Figure S3. Immunoblotting of renal tissues in mice

The full-length of immunoblots presented in Figure 5b. Control mice, lane 1-4; STZ-Vehicle mice, lane 7-10; STZ-MSC mice, lane 12-16.

Supplementary Table S1. Primary antibodies

Abs (P/M)	Species	Reactivity	Manufacturer
**Immunofluorescence / Immunohistochemistry**			
F4/80 (P)	Gt	Hu, Ms, Rt	Santa Cruz Biotechnology, Inc., Dallas, TX
ICAM-1 (M)	Ms	Ms	BioLegend, Inc., San Diego, CA
TNF-α (P)	Rb	Hu, Ms, Chk, GP	Abcam, Inc., Cambridge, UK
Megalin (P)	Gt	Ms, Rt	Santa Cruz Biotechnology, Inc., Dallas, TX
TGF-β1 (P)	Gt	Hu, Ms	Santa Cruz Biotechnology, Inc., Dallas, TX
TGF-β1(P)	Rb	Ms, Rt, Hu, Pg	Abcam, Cambridge, UK
ZO-1 (M)	Rt	Ms	Abcam, Cambridge, UK
ZO-1(P)	Rb	Hu, Ms, Rt, Dg	Invitrogen, Carlsbad, CA
Lectin (LTL)-FITC conjugated			EY Laboratories, Inc., San Mateo, CA
**Immunoblotting**			
HSP70	Rb	Hu	System Biosciences, Inc., Mountain View, CA
CD9	Rb	Hu	System Biosciences, Inc., Mountain View, CA
CD63	Rb	Hu	System Biosciences, Inc., Mountain View,

			CA
β-actin	Ms	Hu, B, Sh, Pg, Rb, Ct, Dg, Ms, Rt, GP, Chk, Cp	Sigma-Aldrich, St. Louis, MO
p38-MAPK	Rb	Hu, Ms, Rt, Mk, GP	Cell Signaling Technology, Inc. Danvers, MA
p-p38-MAPK	Rb	Hu, Ms, Rt, Mk, Dm, Pg, Sc	Cell Signaling Technology, Inc. Danvers, MA

Supplementary Table S2. Secondary antibodies

Abs	Species	Conjugate	Manufacturer
**Immunoblotting**			
Rabbit IgG	Gt	HRP	Amersham, Little Chalfont, UK
Mouse IgG	Sh	HRP	Amersham, Little Chalfont, UK
**Immunofluorescence**			
Rabbit IgG	Gt	Cy3	Millipore, Billerica, MA
Mouse IgG	Dnk	Cy3	Millipore, Billerica, MA
Goat IgG	Dnk	Cy3	Millipore, Billerica, MA

Supplementary Figure S1

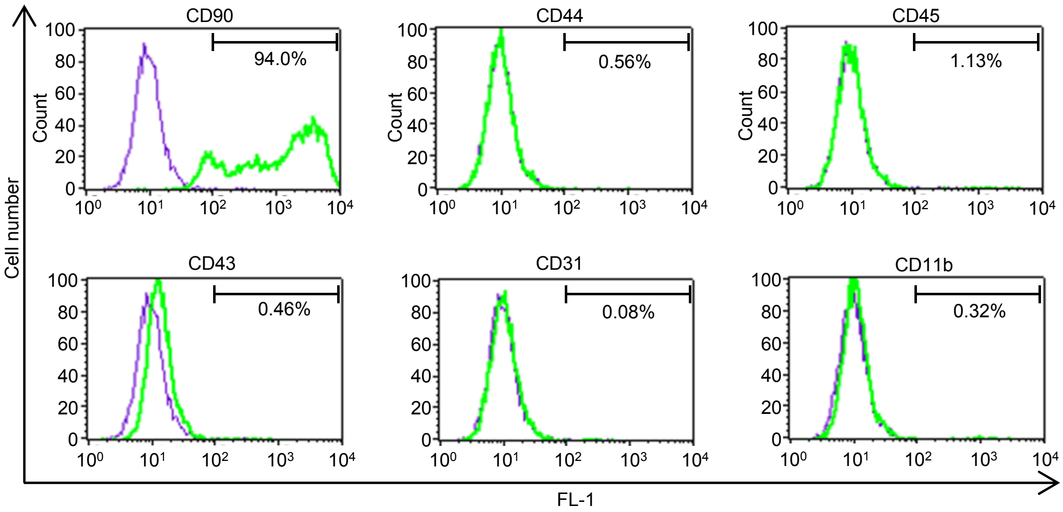

Supplementary Figure S1. Characterization of rat MSCs

Flow cytometry analysis of the expression of cell surface markers related to rat MSCs. Green line shows the population which is stained with target antibody. Purple line shows the population which is stained with isotype control antibody.

Supplementary Figure S2

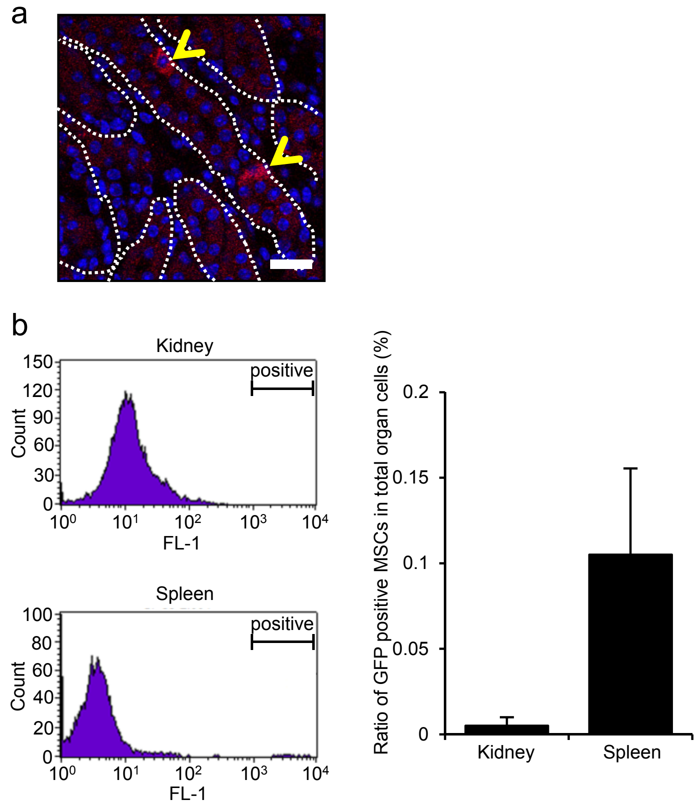

Supplementary Figure S2. Distribution of rat MSCs in diabetic mice

(a) Distribution of MSCs marked with PKH26 in the kidney of HFD mice at 1 week after the initial MSC injection is shown in the panel. Bar: 20 µm. (b) Distribution of GFP-positive MSCs in the kidney and spleen of STZ mice at 1 week after the initial MSC injection is shown. Flow cytometry analysis of GFP-positive cells is shown in the left panels. The ratio of GFP-positive MSCs in total cells isolated from kidney and spleen is shown in the right panel. Data are expressed as mean ± SE values of 4 animals.

Supplementary Figure S3

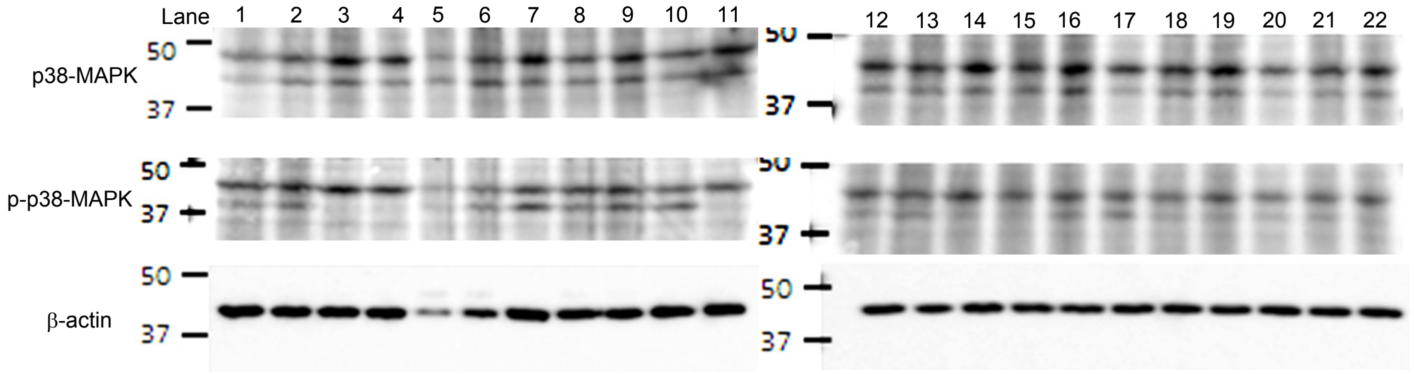

Supplementary Figure S3. Immunoblotting of renal tissues in mice
The full-length blots in Figure 5b. Control mice, lane 1-4; STZ-Vehicle mice, lane 7-10; STZ-MSC mice, lane 12-16.
